# Epidemiology and awareness of hypertension in a rural Ugandan community: a cross-sectional study

**DOI:** 10.1186/1471-2458-13-1151

**Published:** 2013-12-09

**Authors:** Prashant Kotwani, Dalsone Kwarisiima, Tamara D Clark, Jane Kabami, Elvin H Geng, Vivek Jain, Gabriel Chamie, Maya L Petersen, Harsha Thirumurthy, Moses R Kamya, Edwin D Charlebois, Diane V Havlir

**Affiliations:** 1 HIV/AIDS Division, Department of Medicine, San Francisco General Hospital, University of California San Francisco, 995 Potrero Avenue, UCSF Box 0874, San Francisco, California 94110, USA; 2 Makerere University-University of California San Francisco (MU-UCSF) Research Collaboration, Mbarara, Uganda; 3 Mulago Joint AIDS Program, Kampala and Mbarara, Mbarara, Uganda; 4 School of Public Health, University of California, Berkeley, California, USA; 5 Gillings School of Global Public Health, University of North Carolina at Chapel Hill, Chapel Hill, North Carolina, USA; 6 Department of Medicine, School of Medicine, Makerere University College of Health Sciences, Kampala, Uganda; 7 Center for AIDS Prevention Studies, Department of Medicine, University of California San Francisco, San Francisco, California, USA

**Keywords:** Hypertension, Epidemiology, Blood pressure, Non-communicable disease, Community health, Health campaign, Public health, Sub-Saharan Africa, Uganda, Rural

## Abstract

**Background:**

Hypertension is one of the largest causes of preventable morbidity and mortality worldwide. There are few population-based studies on hypertension epidemiology to guide public health strategies in sub-Saharan Africa. Using a community-based strategy that integrated screening for HIV and non-communicable diseases, we determined the prevalence, awareness, treatment rates, and sociodemographic factors associated with hypertension in rural Uganda.

**Methods:**

A household census was performed to enumerate the population in Kakyerere parish in Mbarara district, Uganda. A multi-disease community-based screening campaign for hypertension, diabetes, and HIV was then conducted. During the campaign, all adults received a blood pressure (BP) measurement and completed a survey examining sociodemographic factors. Hypertension was defined as elevated BP (≥140/≥90 mmHg) on the lowest of three BP measurements or current use of antihypertensives. Prevalence was calculated and standardized to age distribution. Sociodemographic factors associated with hypertension were evaluated using a log-link Poisson regression model with robust standard errors.

**Results:**

Community participation in the screening campaign was 65%, including 1245 women and 1007 men. The prevalence of hypertension was 14.6%; awareness of diagnosis (38.1%) and current receipt of treatment (20.6%) were both low. Age-standardized to the WHO world standard population, hypertension prevalence was 19.8%, which is comparable to 21.6% in the US and 18.4% in the UK. Sociodemographic factors associated with hypertension included increasing age, male gender, overweight, obesity, diabetes, alcohol consumption, and family history. Prevalence of modifiable factors was high: 28.3% women were overweight/obese and 24.1% men consumed ≥10 alcoholic drinks per month.

**Conclusions:**

We found a substantial burden of hypertension in rural Uganda. Awareness and treatment of hypertension is low in this region. Enhanced community-based education and prevention efforts tailored to addressing modifiable factors are needed.

## Background

Increasing urbanization has fueled social and economic changes in sub-Saharan Africa, which have contributed to a surge in non-communicable disease (NCD), including hypertension [1,2]. High blood pressure is the leading risk factor for mortality worldwide and approximately 80% of all cardiovascular deaths occur in low- and middle-income countries [3-5]. It is predicted that more than 125 million adults in sub-Saharan Africa alone will have hypertension by 2025 [6,7]. Given the long-term decreased productivity associated with hypertension, identifying and treating a large proportion of patients has the potential to generate tremendous social and economic benefits in this region [8-13].

Despite the recognition of hypertension as a major threat to public health in sub-Saharan Africa, epidemiologic research to guide public health approaches to prevent and treat hypertension is limited in scope, sample size, methodology, and generalizability of conclusions [7,8,12,14-16]. Robust population-based data using sound statistics are still needed to confirm prior estimates and inform policy debates. Further, strategies that allow hypertension screening on a population level need to be developed in these resource-limited settings.

Novel community- and home-based testing programs are being deployed as a means to diagnose and treat HIV in sub-Saharan Africa [17,18]. These programs address barriers such as high cost of accessing clinic-based testing, inadequate primary care services and lack of awareness about testing. Leveraging this HIV targeted infrastructure can improve services for NCDs, especially in the context of a fragile health care delivery system [12,19-22]. Building these strategic partnerships can also be used to study the epidemiology of NCDs.

The Sustainable East Africa Research in Community Health (SEARCH) collaboration has pioneered multi-disease prevention and treatment services that integrate HIV and NCD services through community-based screening campaigns. In a prior pilot campaign conducted in May 2011 in a rural community of southwestern Uganda, we found a significant burden of untreated hypertension [23]. In the present study, we sought to determine the prevalence, awareness, treatment rates and sociodemographic factors associated with hypertension in this community using an approach that integrates HIV and NCD testing.

## Methods

### Study setting

The study was conducted in Kakyerere parish within Mbarara district in southwestern Uganda. A parish is an administrative unit in Uganda that consists of multiple villages. Kakyerere parish includes 9 villages comprising approximately 6,500 persons with 50% of the population ≥18 years [23].

### Community census

A community-wide household level census was performed in Kakyerere parish. Trained staff, with the help of local leaders, identified and attempted to visit all households within the boundary of the parish. When a visit was made, the head of the household provided informed consent to collect demographic information about all household members. A unique household-identifier was given to each household, which was used to accurately identify participants at the community health campaign. The census allowed ascertainment of community participation in the campaign, understanding of basic sociodemographics of non-participants, and calculation of age-standardized measures for the whole parish population.

### Community health campaign

Community health campaigns (CHC) are large public health initiatives that provide rapid diagnostic services for multiple diseases in rural areas. Prior to the campaign, local leaders designed and executed community mobilization measures including radio announcements and posters in order to maximize participation at the CHC. Local health personnel staffed each campaign and provided services for adults and children at community sites such as schools and churches. The CHC occurred over five days and each adult participant received point-of care screening for HIV, hypertension, and diabetes. Those screening positive for any disease were given a follow-up appointment at a local health facility for further management. Children were offered HIV testing, rapid diagnosis and treatment for malaria, and deworming. Each person who joined in the CHC participated on one campaign day for 2–3 hours. The campaign is “high throughput” because it provides rapid services for a large population (approximately 1,000 persons/day). Detailed procedures regarding the implementation of a CHC in Kakyerere parish have been recently described by our group [23].

### Blood pressure measurement

All adult CHC participants received blood pressure (BP) screening and completed an epidemiologic survey at the CHC. Inclusion criteria were age ≥18 years and residence in Kakyerere parish. Hypertension was defined as systolic BP ≥140 mmHg or diastolic BP ≥90 mmHg per the World Health Organization (WHO)/International Society of Hypertension (ISH) guidelines [24]. Hypertension was further classified as Stage 1 (140-159/90-99 mmHg) or Stage 2 (≥160/≥100 mmHg) based on the Seventh report of the Joint National Committee on prevention, detection, evaluation, and treatment of high blood pressure (JNC7) guidelines [25].

Trained staff conducted BP measurements using an electronic, automated sphygmomanometer (Honsun LD7A), with small (<21 cm), normal (22–32 cm), and large (>33 cm) cuff sizes. Subjects were seated in a chair and rested for approximately one minute before BP was measured. Following initial BP measurement, participants who met criteria for hypertension during this screen underwent two repeat measurements one minute apart. The lowest of three measurements was used to establish a diagnosis of hypertension.

Height was measured to the nearest 0.1 cm after removal of shoes. Weight was measured to the nearest kilogram after removal of shoes and heavy clothing. Body mass index (BMI) was calculated to determine proportion of overweight (25.0-29.9 kg/m^2^) and obese (≥30 kg/m^2^) participants. In addition, point-of-care random plasma glucose (Optium Xeed - Abbot) and HIV rapid tests (Determine HIV-1/HIV-2 (Abbot); HIV-1/2 Stat-Pak assay; and Uni-Gold HIV Rapid test) were also performed. Trained staff conducted a survey to determine sociodemographic factors and treatment history for hypertension.

### Hypertension treatment referral

Hypertensive adults identified at the CHC were given a referral appointment to a local government health facility, where they received treatment per the Ugandan Ministry of Health guidelines [26]. Government health facilities in Uganda are required to offer free services to all persons, including supply of antihypertensive medications. In the event of antihypertensive drug shortages at government facilities, patients are occasionally referred to private retail pharmacies to obtain antihypertensive medications. In a previous report, the monthly cost of purchasing antihypertensives has been estimated at 1.1 Euros (1.4 US Dollars) in a similar region of sub-Saharan Africa [27].

### Data analysis

Survey data were collected via a computerized interview programmed onto data tablets. Stata v.12 (Stata Corporation) was used for all analyses.

Hypertension was defined as systolic BP ≥140 mmHg or diastolic BP ≥90 mmHg on lowest of three BP measures or current use of antihypertensives. Age-standardized prevalence of hypertension was calculated using the Kakyerere parish community-wide census population, 2000 US census population [28], and WHO world standard population [29]. Awareness of a diagnosis of hypertension was defined by self-report among those determined to be hypertensive. BP control was defined as having BP below hypertension threshold (systolic BP < 140 and diastolic BP < 90 mmHg) if currently taking antihypertensives.

We conducted a sensitivity analysis on our prevalence estimate to account for transient changes in BP levels. The upper estimate was calculated by including those with self-reported hypertension but normal BP on screening. The lower estimate was calculated by using a stricter definition of hypertension, systolic BP ≥160 or diastolic BP ≥95 mm Hg.

Sociodemographic factors that may be associated with hypertension prevalence were analyzed. Specifically, we evaluated age, gender, BMI, level of education, occupation, smoking status, alcohol consumption, family history, and random plasma glucose. Due to well-established links between HIV and cardiovascular complications [30], we also assessed HIV status.

Associations between sociodemographic factors and hypertension were investigated using a multivariable log-link Poisson model with robust standard errors to estimate adjusted prevalence ratios. All sociodemographic factors were included *a priori* in the multivariable regression model. Analyses were stratified by gender.

### Ethics

The study was reviewed and approved by the Makerere University School of Medicine Research and Ethics Committee, the Ugandan National Council on Science and Technology, and the UCSF Committee on Human Research.

## Results

### Characteristics of study participants

2,282 adults attended the CHC and 2,252 (99%) were screened for hypertension and answered epidemiologic survey at the CHC. These persons represented 65% of the 3,485 adults (1,861 women and 1,624 men) enumerated in the Kakyerere parish community census. Participation in the CHC was higher among women (66.9%) than men (62.0%) (P = 0.002) (Table 1). Participation was lowest among 18–29 year olds for both women (56.6%, P < 0.0001) and men (58.4%, P = 0.005).

**Table 1:** Number of community members screened for hypertension at the community health campaign (CHC) in Kakyerere parish, in categories of age and gender

		
**Women:**		
**Age**	**Persons screened**	**Community participation (%)**
18–29	478	56.6
30–44	415	75.4
45–59	194	75.8
≥60	158	75.2
Total	1245	66.9
**Men:**		
**Age**	**Persons screened**	**Community participation (%)**
18–29	448	58.4
30–44	315	62.4
45–59	151	68.9
≥60	93	69.4
Total	1007	62.0

Demographic and clinical characteristics are listed in Table 2.

**Table 2:** Demographic and clinical characteristics of study participants

	**Women**	**Men**	**Total**
	**(N = 1245)**	**(N = 1007)**	**(N = 2252)**
		**N (%)**	
**Age (years)**			
18–29	478 (38.4%)	448 (44.5%)	926 (41.1%)
30–44	415 (33.3%)	315 (31.3%)	730 (32.4%)
45–59	194 (15.6%)	151 (15.0%)	345 (15.3%)
≥ 60	158 (12.7%)	93 (9.2%)	251 (11.2%)
**Marital status**^**a**^			
Single	172 (14.6%)	300 (32.7%)	472 (22.5%)
Married (ever)	1009 (85.4%)	618 (67.3%)	1627 (77.5%)
**BMI (kg/m**^**2**^**)**^**b**^			
≤ 25	892 (71.7%)	923 (91.7%)	1815 (80.6%)
25 – 30	243 (19.5%)	68 (6.8%)	311 (13.8%)
≥ 30	110 (8.8%)	15 (1.5%)	125 (5.5%)
**Occupation**			
Unemployed	158 (12.7%)	176 (17.5%)	334 (14.8%)
Manual	1014 (81.4%)	758 (75.3%)	1772 (78.7%)
Sedentary	73 (5.9%)	73 (7.2%)	146 (6.5%)
**Education level**			
No school	310 (24.9%)	94 (9.3%)	404 (17.9%)
Primary	651 (52.3%)	532 (52.8%)	1183 (52.5%)
Secondary	222 (17.8%)	297 (29.5%)	519 (23.1%)
Tertiary and beyond	62 (5.0%)	84 (8.3%)	146 (6.5%)
**Acres of land owned**^**c**^			
None	142 (12.4%)	96 (10.2%)	238 (11.4%)
≤ 1	282 (24.5%)	157 (16.6%)	439 (21.0%)
1–5	484 (42.1%)	370 (39.2%)	854 (40.8%)
≥ 5	242 (21.0%)	322 (34.1%)	564 (26.9%)
**Current tobacco use**			
No	1208 (97.0%)	783 (77.8%)	1991 (88.4%)
Yes	37 (3.0%)	224 (22.2%)	261 (11.6%)
**Alcohol use (drinks consumed per month)**^**d**^			
None	986 (79.3%)	527 (52.4%)	1513 (67.3%)
0–10	196 (15.7%)	236 (23.5%)	432 (19.2%)
≥ 10	62 (5.0%)	242 (24.1%)	304 (13.5%)
**HIV**			
Negative	1107 (88.9%)	923 (91.7%)	2030 (90.1%)
Positive	138 (11.1%)	84 (8.3%)	222 (9.9%)
**Random plasma glucose (mmol/L)**^**e**^			
0–7	1101 (88.7%)	895 (89.2%)	1996 (88.9%)
7–11	126 (10.2%)	103 (10.3%)	229 (10.2%)
≥ 11.1	14 (1.1%)	5 (0.5%)	19 (0.9%)
**Family history**^**f**^			
No	962 (79.5%)	798 (81.6%)	1760 (80.4%)
Yes	248 (20.5%)	180 (18.4%)	428 (19.6%)
**Blood pressure (BP) (mm Hg)**			
Systolic Blood Pressure Mean (SD)	122.5 (18.6)	125.5 (16.7)	123.8 (17.9)
Diastolic Blood Pressure Mean (SD)	74.9 (12.1)	74.8 (11.7)	74.9 (11.9)
**Hypertension** (elevated BP or taking antihypertensives)	192 (15.4%)	162 (16.1%)	354 (15.7%)
**BP ≥140/≥90 mm Hg**	170 (13.7%)	155 (15.4%)	325 (14.4%)
Stage 1 (140–159/90–99)	104 (8.4%)	108 (10.7%)	212 (9.4%)
Stage 2 (≥160/≥100)	66 (5.3%)	47 (4.7%)	113 (5.0%)
**Self-reported hypertension**	164 (13.2%)	60 (6.0%)	224 (10.0%)
**Awareness (if hypertensive, N = 354)**	97 (50.5%)	38 (23.5%)	135 (38.1%)
**Receiving treatment (if hypertensive, N = 354)**	55 (28.6%)	18 (11.1%)	73 (20.6%)
**BP controlled (if receiving treatment, N = 73)**	22 (40.0%)	7 (38.9%)	29 (39.7%)

^a^N = 2099, female = 1181, male = 918.

^b^N = 2251, female = 1245, male = 1006.

^c^N = 2095, female = 1150, male = 945.

^d^N = 2249, female = 1244, male = 1005.

^e^N = 2244, female = 1241, male = 1003.

^f^N = 2188, female = 1210, male = 978.

### Prevalence, awareness, treatment and control of hypertension

The overall unadjusted prevalence of hypertension in this community was 15.7%. Hypertension prevalence was 16.1% among men and 15.4% in women (P = 0.67) (Table 2). Per JNC7 classification [25], 9.4% participants had Stage 1 hypertension while 5.0% had Stage 2 hypertension.

A large fraction of patients were not aware of being hypertensive and received a new diagnosis at the CHC. Awareness was higher among women: 50.5% of hypertensive women were aware of their diagnosis compared to only 23.5% men (P < 0.0001). Self reported use of antihypertensives was also significantly more common among women (28.6%) compared to men (11.1%) (P < 0.0001). Among those using antihypertensive medication, a similar proportion of women (40.0%) and men (38.9%) achieved BP control (normotensive on reported therapy) (P = 0.93).

### Age-standardized prevalence

Using the Kakyerere parish household census population age distribution, the age-standardized prevalence for the entire community was 14.6%, (95% confidence interval (CI) 13.3-15.9; with women at 13.4%, and men at 16.0%). Using the 2000 US census population and the WHO world standard population, the age-standardized prevalence was 22.0% (95% CI 20.0-24.0) and 19.8% (95% CI 18.0-21.5), respectively (Table 3).

**Table 3:** Age-standardized prevalence of hypertension using the Kakyerere Parish household census population, the 2000 U.S. standard population, and the WHO world standard population among adults ≥18 years

	**Prevalence (95% CI)**			
	**Study participants (unadjusted)**	**Kakyerere Parish household census population**	**2000 US census population**	**WHO world standard population**
**Total**	15.7% (14.2–17.2)	14.6% (13.3–15.9)	22.0% (20.0–24.0)	19.8% (18.0–21.5)
**Women**	15.4% (13.4–17.4)	13.4% (11.7–15.1)	21.1% (18.5–23.6)	18.7% (16.5–21.0)
**Men**	16.1% (13.8–18.4)	16.0% (13.9–18.2)	23.1% (19.9–26.2)	21.0% (18.2–23.8)

### Sociodemographic factors associated with hypertension

Among women, significant associations with hypertension in the adjusted regression model included age, BMI, and diabetes (random plasma glucose ≥11.1 mmol/L) (Table 4). Age ≥60 years was associated with a marked elevation in hypertension prevalence compared to age 18–29 years (PR = 12.30; 95% CI 6.59-22.10). Similarly, the prevalence of hypertension increased with BMI: overweight and obese women were more likely to be hypertensive (PR = 1.75; 95% CI 1.28-2.40, and PR = 2.72; 95% CI 1.94-3.83, respectively). Diabetes was also associated with substantially increased likelihood of being hypertensive (PR = 2.44; 95% CI 1.32-4.51).

**Table 4:** Risk factors associated with hypertension using a log-link Poisson model with robust standard errors

	**Women (N = 1245**^**a**^**)**			**Men (N = 1007**^**a**^**)**		
	**Proportion with hypertension (%)**	**Unadjusted prevalence ratio**	**Adjusted**^**b **^**prevalence ratio**	**Proportion with hypertension**	**Unadjusted prevalence ratio**	**Adjusted**^**b **^**prevalence ratio**
		**PR (95% CI)**	**PR (95% CI)**	**(%)**	**PR (95% CI)**	**PR (95% CI)**
**Age (years)**		*P < 0.0001*	***P < 0.0001***		*P < 0.0001*	***P < 0.0001***
18–29	16/478 = 3.4%	1	1	29/326 = 6.5%	1	1
30–44	58/415 = 14.0%	4.18 (2.44–7.15)	3.93 (2.15–7.18)	50/315 = 15.9%	2.45 (1.59–3.79)	2.13 (1.10–4.11)
45–59	48/194 = 24.7%	7.39 (4.30–12.70)	6.20 (3.33–11.55)	44/151 = 29.1%	4.50 (2.92–6.93)	3.94 (2.05–7.56)
≥60	70/158 = 44.3%	13.24 (7.93–22.10)	12.30 (6.59–22.96)	39/93 = 41.9%	6.48 (4.23–9.92)	6.00 (3.10–11.58)
**Marital Status**		*P = 0.006*	*P = 0.35*		*P < 0.0001*	*P = 0.94*
Single	14/172 = 8.1%	1	1	23/300 = 7.7%	1	1
Married (ever)	171/1009 = 16.9%	2.08 (1.24–3.50)	0.79 (0.48–1.30)	132/618 = 21.4%	2.79 (1.83–4.24)	1.03 (0.53–2.01)
**BMI (kg/m**^**2**^**)**		*P < 0.0001*	***P < 0.0001***		*P < 0.0001*	***P = 0.0004***
< 25	105/892 = 11.8%	1	1	130/923 = 14.1%	1	1
25–30	48/243 = 19.7%	1.68 (1.23–2.29)	1.75 (1.28–2.40)	24/68 = 35.3%	2.51 (1.75–3.59)	2.12 (1.45–3.11)
>30	39/110 = 35.5%	3.01 (2.21–4.1)	2.72 (1.94–3.83)	7/15 = 46.7%	3.31 (1.89–5.83)	1.64 (0.84–3.22)
**Occupation**		*P = 0.99*	*P = 0.36*		*P = 0.06*	*P = 0.92*
Unemployed	24/158 = 15.2%	1	1	20/176 = 11.4%	1	1
Manual	157/1014 = 15.5%	1.02 (0.69–1.51)	0.79 (0.53–1.18)	125/758 = 16.5%	1.45 (0.93–2.26)	1.06 (0.67–1.69)
Sedentary	11/73 = 15.1	0.99 (0.51–1.92)	1.09 (0.57–2.10)	17/73 = 23.3%	2.05 (1.14–3.68)	1.16 (0.57–2.35)
**Education**		*P < 0.0001*	*P = 0.61*		*P = 0.05*	*P = 0.43*
No school	80/310 = 25.8%	2.05 (1.55–2.70)	1.19 (0.88–1.62)	19/94 = 20.2%	1.25 (0.80–1.95)	1.15 (0.73–1.80)
Primary	82/651 = 12.6%	1	1	86/532 = 16.2%	1	1
Secondary	23/222 = 10.4%	0.82 (0.53–1.27)	1.22 (0.77–1.94)	37/297 = 12.5%	0.77 (0.54–1.10)	1.03 (0.70–1.50)
Tertiary and beyond	7/62 = 11.3%	0.90 (0.43–1.85)	1.00 (0.44–2.28)	20/84 = 23.8%	1.47 (0.96–2.26)	1.52 (0.90–2.57)
**Acres of land owned**		*P = 0.50*	*P = 0.56*		*P = 0.58*	*P = 0.90*
None	17/142 = 12.0%	1	1	11/96 = 11.5%	1	1
≤ 1	50/282 = 17.7%	1.48 (0.89–2.47)	1.47 (0.86–2.52)	27/157 = 17.2%	1.50 (0.78–2.88)	1.03 (0.54–1.98)
1–5	76/484 = 15.7%	1.31 (0.80–2.14)	1.30 (0.78–2.18)	64/370 = 17.3%	1.51 (0.83–2.75)	1.17 (0.65–2.09)
≥ 5	40/242 = 16.5%	1.38 (0.81–2.34)	1.24 (0.72–2.14)	51/322 = 15.8%	1.38 (0.75–2.54)	1.07 (0.59–1.93)
**Tobacco use**		*P = 0.54*	*P = 0.49*		*P = 0.22*	*P = 0.70*
No	185/1208 = 15.3%	1	1	120/783 = 15.3%	1	1
Yes	7/37 = 18.9%	1.24 (0.63–2.44)	0.78 (0.39–1.57)	42/224 = 18.8%	1.22 (0.89–1.68)	0.94 (0.67–1.31)
**Alcohol use**^**c**^		*P = 0.70*	*P = 0.33*		*P < 0.0001*	***P = 0.02***
None	148/986 = 15.0%	1	1	59/527 = 11.2%	1	1
0–10	34/196 = 17.4%	1.16 (0.82–1.62)	0.92 (0.66–1.28)	38/236 = 16.1%	1.44 (0.99–2.10)	1.17 (0.79–1.71)
≥10	10/62 = 16.1%	1.07 (0.60–1.93)	0.58(0.28–1.21)	64/242 = 26.5%	2.36 (1.72–3.25)	1.60 (1.13–2.26)
**HIV**		*P = 0.005*	*P = 0.24*		*P = 0.10*	***P = 0.02***
Negative	183/1107 = 16.5%	1	1	154/923 = 16.7%	1	1
Positive	9/138 = 6.5%	0.39 (0.21–0.75)	0.69 (0.37–1.28)	8/84 = 9.5%	0.57 (0.29–1.12)	0.39 (0.17–0.87)
**RPG**^**d**^		*P < 0.0001*	***P = 0.02***		*P = 0.0002*	*P = 0.75*
< 7	160/1101 = 14.5%	1	1	135/895 = 15.1%	1	1
7 – 11	23/126 = 18.3%	1.26 (0.84–1.87)	1.02 (0.68–1.52)	24/103 = 23.3%	1.54 (1.05–2.27)	1.17 (0.78–1.76)
≥ 11.1	8/14 = 57.1%	3.93 (2.44–6.33)	2.44 (1.32–4.51)	3/5 = 60.0%	3.98 (1.91–8.28)	1.10 (0.58–2.09)
**Family history**		*P = 0.11*	*P = 0.11*		*P = 0.002*	***P = 0.007***
No	139/962 = 14.5%	1	1	114/798 = 14.3%	1	1
Yes	46/248 = 18.6%	1.28 (0.95–1.74)	1.26 (0.95–1.69)	42/180 = 23.3%	1.63 (1.19–2.24)	1.51 (1.12–2.04)

^a^1067 women and 839 men included in the multivariate regression model.

^b^Each factor is adjusted for all other factors in the table.

^c^Drinks of alcohol consumed per month.

^d^RPG – Random plasma glucose in mmol/L.

Among men, sociodemographic predictors of hypertension included age, BMI, alcohol use, HIV status, and family history. Increasing age was associated with an increase in the prevalence of hypertension: older men (≥60 years) were six times as likely to have hypertension compared to younger men (18–29 years), PR = 6.00 (95% CI 3.10-11.58). Increase in BMI was also associated with a greater hypertension prevalence: PR = 2.12 (95% CI 1.45-3.11) for overweight men and PR = 1.64 (95% CI 0.84-3.22) for obese men. Men consuming ≥10 alcoholic drinks per month had 60% higher prevalence of hypertension compared to non-drinkers, PR = 1.60 (95% CI 1.13-2.26). HIV infected men were less likely to be hypertensive, PR = 0.39 (95% CI 0.17-0.87). Men with a family history of hypertension in first degree relatives were 1.5 times as likely to have hypertension than those without a family history, PR = 1.51 (95% CI 1.12-2.04).

In a separate multivariable model including gender, men were 1.3 times more likely to have hypertension than women after adjustment for all other variables, PR = 1.34 (95% CI 1.05-1.69) (data not shown).

### Sensitivity analysis

Estimated prevalence of hypertension was 19.7% (women 20.8% and men 18.3%) if those with self-reported hypertension were considered. A lower estimate of 6.6% (women 6.7% and men 6.4%) was obtained by using more stringent BP measures (SBP ≥160 or DBP ≥ 95 mm Hg).

## Discussion

Using a community-based strategy that integrated HIV and NCD screening, we found a substantial burden of hypertension (14.6% age-standardized prevalence) accompanied by low rates of awareness of the condition. Among individuals aware of their hypertension, we found low rates of therapy. Furthermore, we found a high prevalence of multiple modifiable factors associated with hypertension. Our study, by leveraging a multi-disease CHC to study hypertension, demonstrates that strategic public health investments in HIV and NCDs can be complementary and efficient in providing diagnostic services in Sub-Saharan Africa.

Using this community-based approach we provide estimates of hypertension prevalence in the range of those previously reported from Uganda [12,31] and sub-Saharan Africa [7]. Age-standardized to the WHO world standard population, hypertension prevalence in Kakyerere parish was 19.8%, which is comparable to 21.6% in the US and 18.4% in the UK [32]. This finding adds to accumulating evidence that prevalence of hypertension in sub-Saharan Africa is approaching that in developed nations [6,32-35]. Further, our data are especially concerning when considering that rural areas in Sub-Saharan Africa have a lower prevalence of hypertension compared to semi-urban and urban areas [16,36-39].

Awareness of a hypertension diagnosis (38.1%) was inadequate and especially poor among men (23.5%). In a review, Addo et al. [11] report that hypertension awareness in sub-Saharan Africa was generally below 40%, consistent with our results. Despite being aware, however, only one out of five known hypertensives was receiving treatment. Further, BP control was achieved in only 39.7% of hypertensive patients on therapy. Addressing these gaps requires health system strengthening. Community health campaigns can be a key component of new strategies that build both individual and community level awareness of NCDs such as hypertension.

Sociodemographic factor analysis identified groups of persons most likely to be hypertensive - these persons can serve as the target for prevention strategies in rural Uganda. Further, modifiable factors associated with hypertension in this population include overweight, obesity, alcohol consumption, and diabetes—all consistent with prior reports [9,12,15,31,40]. BMI and hypertension had a strong relationship among both women and men, while a relationship with alcohol consumption (≥10 drinks/month) was found in men only. Three out of ten women were either overweight or obese compared to only one out of ten men. 24.1% men consumed ≥10 alcoholic drinks/month compared to only 5% women.

Our data also emphasize the need for greater diagnosis and treatment of diabetes, particularly among women. The small number of men found to have elevated blood glucose prevented meaningful analysis of the link between diabetes and hypertension in men. Consistent with other studies across sub-Saharan Africa [11,12,15,40], hypertension had a strong relationship with both increasing age and male gender. Family history was independently related to hypertension among men but not women. These data can be utilized to stratify risk and inform hypertension treatment decisions in rural Uganda.

Interestingly, HIV infected men were less likely to be hypertensive even after multivariable adjustment, PR = 0.39 (0.17-0.87). Previously, Barnighausen et al. reported a 3.0 mm Hg decrease in systolic BP with HIV infection after adjusting for other variables [41]. Another population-based study from South Africa found that HIV-infected persons had a lower prevalence of hypertension than HIV-uninfected persons (19.5% vs 27.9%, p = 0.001) [42]. A longitudinal, prospective study in South Africa also found decreased risk of high blood pressure with HIV infection [43]. Yet, it has been well established that HIV infection has adverse effects on cardiovascular health [44]. The suggestion of an inverse association between HIV and hypertension should be viewed with caution and further evaluated.

In our prior pilot campaign in Kakyerere parish in 2011, we reported a higher prevalence of hypertension (28%), which included self-reported hypertension. In this study we used a more rigorous definition of hypertension (using lowest of three BP measures or current use of antihypertensives) and did not include self-reported hypertension, which likely accounts for the difference in prevalence estimates between the two studies. Further, many participants reported to be hypertensive in our pilot study in 2011 were subsequently found not to have high BP on repeat screening at local health facilities. This over-reporting from our pilot study urged us to develop the more rigorous definition of hypertension that was implemented in our study.

Limitations of our study included incomplete attendance in the CHC, as community members who did not attend the CHC were not screened for hypertension. Conducting a community-wide census allowed us to identify individuals who did not participate and account for bias resulting from differential participation by age in our prevalence estimate. We believe that our age-adjusted prevalence should be a reasonable estimate of the “true” prevalence, considering that age is the most significant risk factor for hypertension in this community. Measurement error in BP can lead to misclassification of hypertensive status; however, we attempted to minimize random error by taking three BP measurements, which increases the validity of our prevalence estimate.

The use of a multi-disease CHC to integrate services for HIV and other NCDs could be a promising public health approach in sub-Saharan Africa. Competition for limited funds and health care resources between NCDs and infectious diseases has already created a sensitive political issue [8-10]. Our approach avoids costs related to establishing new infrastructure and builds efficiency by using HIV as a platform to develop services for other chronic diseases. HIV is also the first chronic disease that has been successfully treated with high-quality, long-term care in resource-limited settings. Building alliances between HIV care and NCD services will improve and expand care for NCDs, consistent with recent declarations of the UN General Assembly [45].

## Conclusions

There is a significant burden of hypertension in rural Uganda, which is comparable to that in developed nations. Among hypertensive patients, awareness of their condition is poor. And among those diagnosed, therapy rates are extremely low. Prevention efforts should focus on persons most likely to be hypertensive, such as overweight/obese, alcohol consumers, and diabetics. Leveraging community-based HIV testing programs to incorporate NCD services is an efficient use of scarce health care resources in sub-Saharan Africa.

## Abbreviations

NCD: Non-communicable disease; HIV: Human immunodeficiency virus; CHC: Community health campaign; BP: Blood pressure; WHO: World Health Organization; ISH: International Society of Hypertension; JNC7: Seventh report of the Joint National Committee on prevention, detection, evaluation, and treatment of high blood pressure; BMI: Body mass index.

## Competing interests

The authors declare that they have no competing interests.

## Authors’ contributions

Conception, design and data collection: PK, DK, TDC, JK, EHG, VJ, GC, MLP, HT, MRK, EDC, DVH. Data analysis: PK, EHG EDC, DVH. Wrote and edited manuscript: PK, DK, TDC, JK, EHG, VJ, GC, MLP, HT, MRK, EDC, DVH. All authors read and approved the final manuscript.

## Pre-publication history

The pre-publication history for this paper can be accessed here:

http://www.biomedcentral.com/1471-2458/13/1151/prepub
